# Supplementary material for: Triple Negative Breast Cancer Preclinical Therapeutic Management by a Cationic Ruthenium-Based Nucleolipid Nanosystem
Source: Int J Mol Sci. 2023 Mar 30;24(7):6473. doi: 10.3390/ijms24076473 (PMC10094725; doi:10.3390/ijms24076473)
Supplement: Supplementary file 1 [file ijms-24-06473-s001.zip › ijms-2303051-supplementary.pdf]

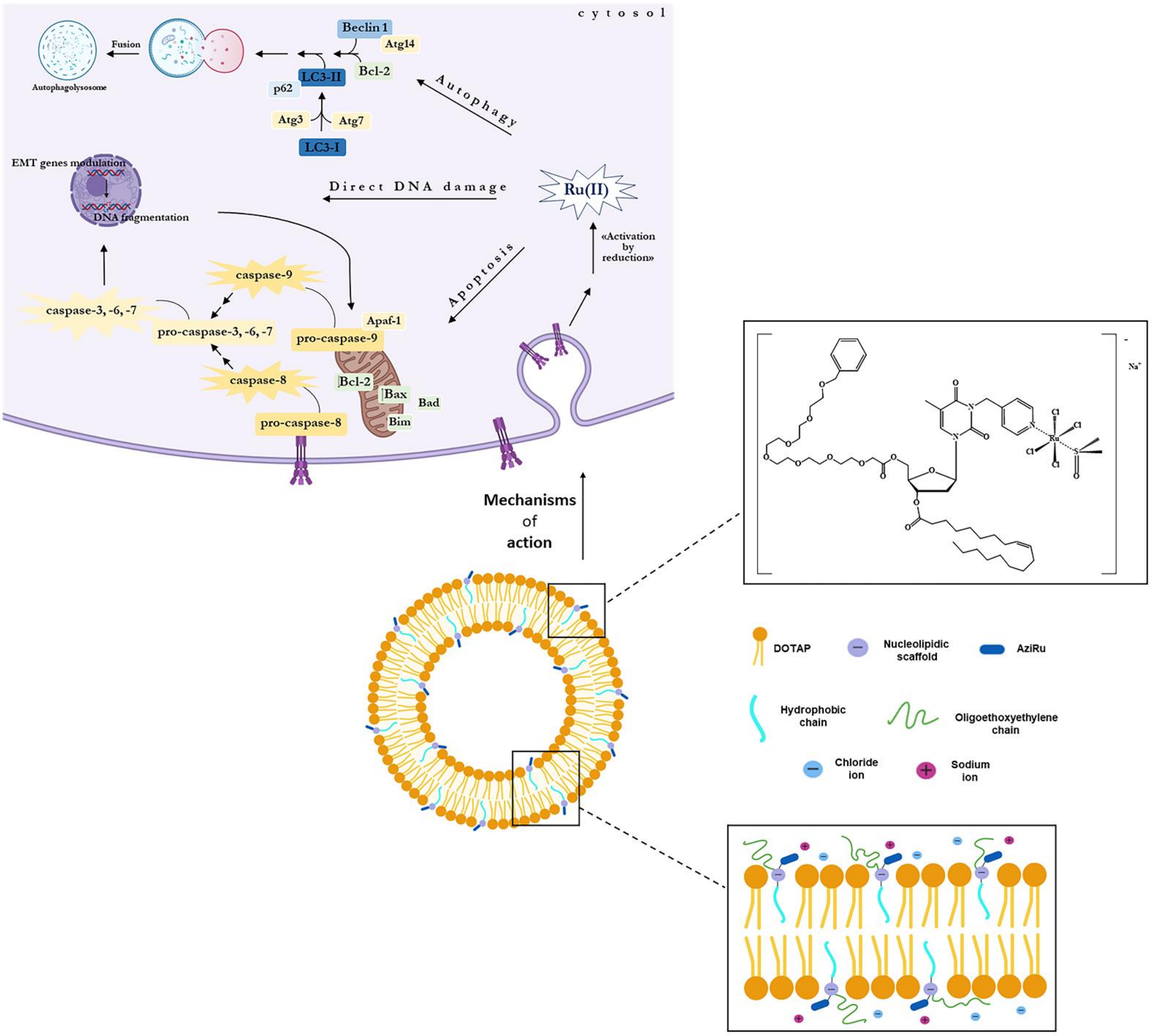

Figure S1. Schematic overview of the liposome HoThyRu/DOTAP molecular composition and its multimodal mechanisms of action in cancer cells.

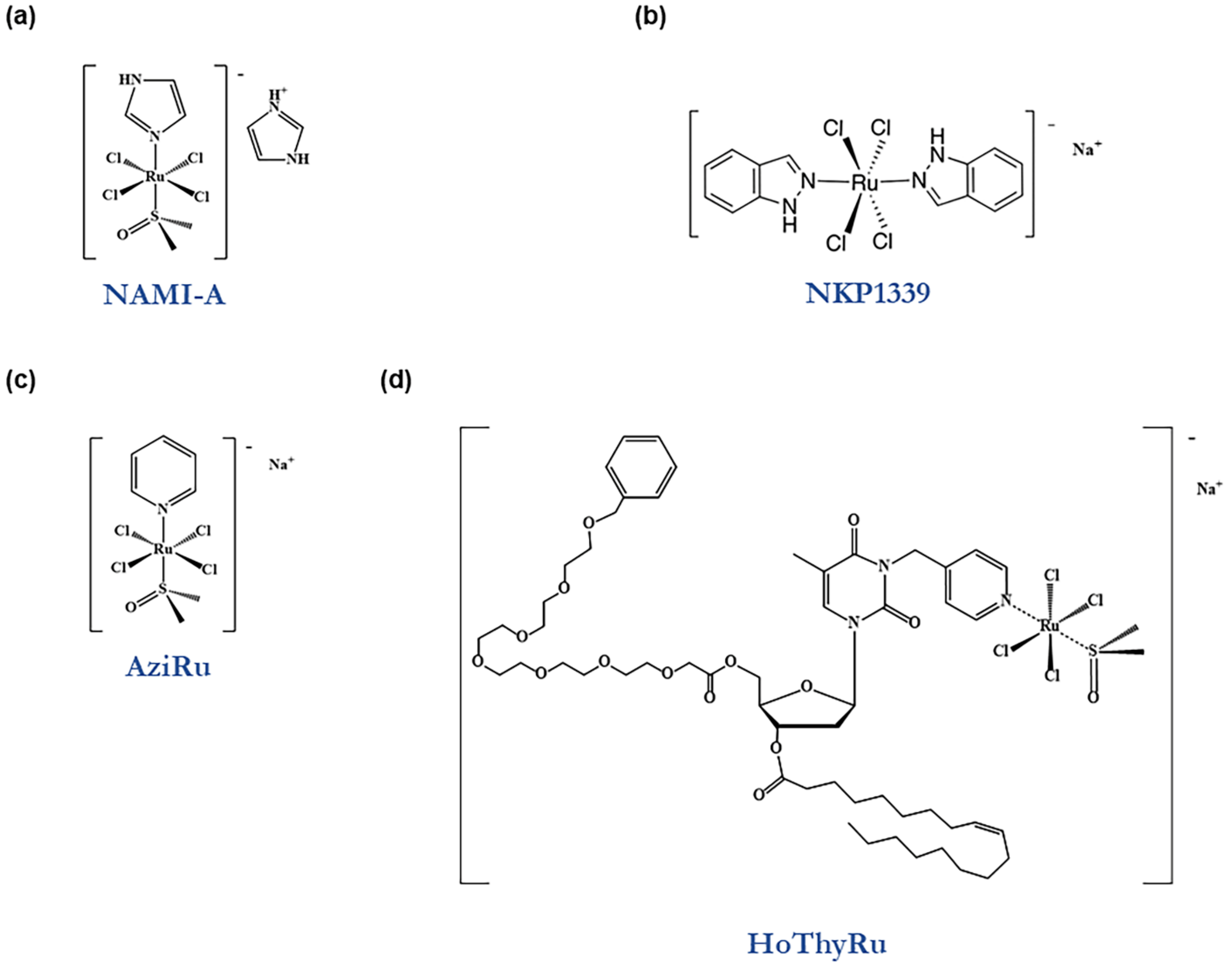

Figure S2. Molecular structures of the low molecular weight ruthenium complexes NAMI-A (**a**), NKP1339 (**b**), AziRu (**c**), and of the HoThyRu (**d**) nucleolipid-based complex incorporating AziRu.

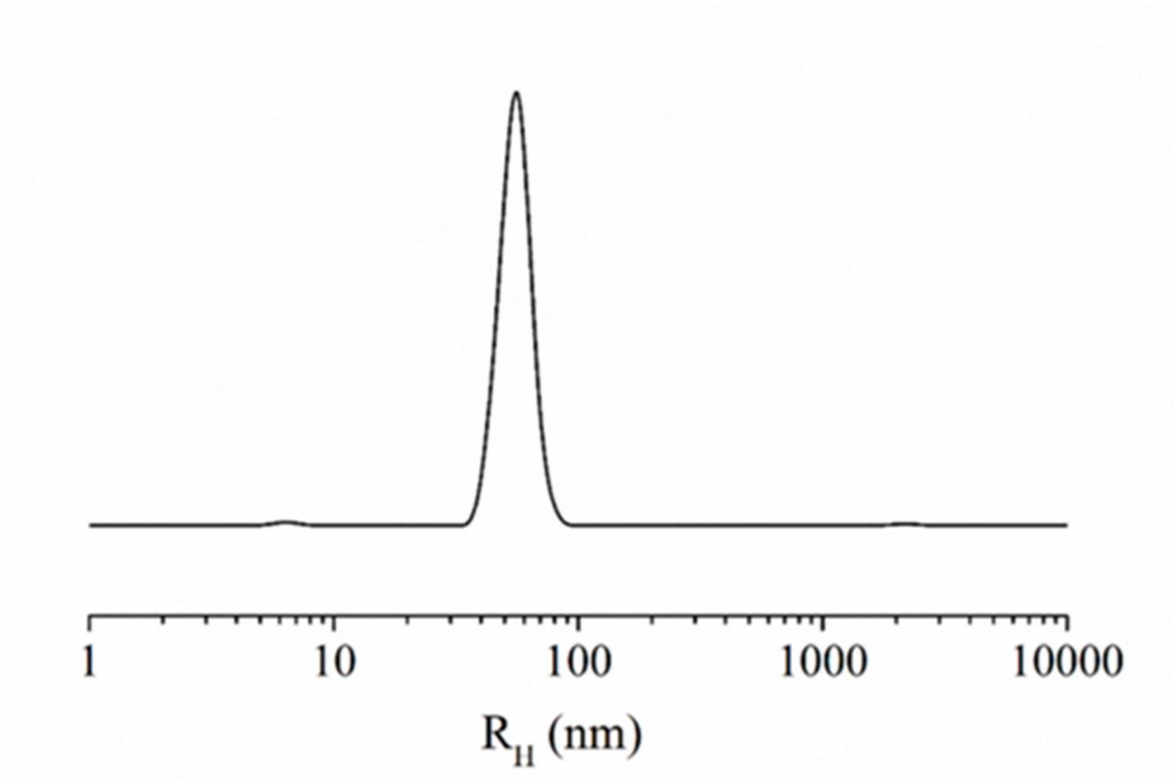

Figure S3. Hydrodynamic radius distribution function obtained through dynamic light scattering (DLS) measurements for the HoThyRu/DOTAP nanoformulations at 30:70 molar ratio.

www.qiagen.com

Quantitation Report

Experiment Information

Run Name	2023-02-17 RU
Run Start	2/17/2023 5:39:47 PM
Run Finish	2/17/2023 6:37:23 PM
Operator	
Notes	
Run On Software Version	Rotor-Gene Q Software 2.3.1.49
Run Signature	The Run Signature is valid.
Gain Green	5.33
Machine Serial No.	0914303

Quantitation Information

Threshold	0.050
Left Threshold	1.000
Standard Curve Imported	No
Standard Curve (1)	N/A
Standard Curve (2)	N/A
Start normalising from cycle	1
Noise Slope Correction	No
No Template Control Threshold	% 0
Reaction Efficiency Threshold	Disabled
Normalisation Method	Dynamic Tube Normalisation
Digital Filter	Light
Sample Page	Page 1
Imported Analysis Settings	

Profile

Cycle	Cycle Point
Hold	Hold @ 95°C, 0min 20s
Cycling (40 repeats)	Step 1: Hold @ 95°C, 1s
	Step 2: Hold @ 60°C, 20s, acquiring to Cycling A([Green][1][1])
Melt	Ramp from 60°C to 95°C
	Hold for 90s on the 1st step
	Hold for 5s on next steps,Melt A([Green][1][1])

Raw Data For Cycling A.Green

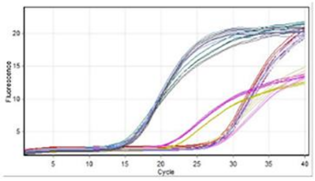

Quantitation data for Cycling A.Green

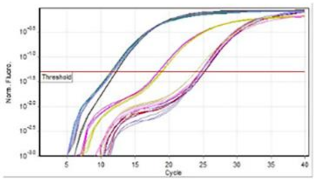

Standard Curve

No.	Color	Name	Type	Ct	Ct Comment	Given Conc (Copies)	Calc Conc (Copies)
1		CTR-E-Cad	Unknown	24.52			
2		CTR-E-Cad	Unknown	24.69			
3		CTR-E-Cad	Unknown	24.55			
4		CTR-N-Cad	Unknown	25.20			
5		CTR-N-Cad	Unknown	24.92			
6		CTR-N-Cad	Unknown	25.12			
7		CTR-Vim	Unknown	11.43			
8		CTR-Vim	Unknown	11.47			
9		CTR-Vim	Unknown	11.59			
10		CTR-Slug	Unknown	18.39			
11		CTR-Slug	Unknown	18.47			
12		CTR-Slug	Unknown	18.46			
13		CTR-Snail	Unknown	23.84			

No.	Color	Name	Type	Ct	Ct Comment	Given Conc (Copies)	Calc Conc (Copies)
14		CTR-Snail	Unknown	23.84			
15		CTR-Snail	Unknown	23.87			
16		CTR-GAPDH	Unknown	11.87			
17		CTR-GAPDH	Unknown	11.87			
18		CTR-GAPDH	Unknown	11.86			
28		Ru-N-Cad	Unknown	24.97			
29		Ru-N-Cad	Unknown	24.83			
30		Ru-N-Cad	Unknown	24.89			
31		Ru-Vim	Unknown	11.39			
32		Ru-Vim	Unknown	11.40			
33		Ru-Vim	Unknown	11.46			
34		Ru-Slug	Unknown	18.96			
35		Ru-Slug	Unknown	18.82			
36		Ru-Slug	Unknown	18.76			
37		Ru-Snail	Unknown	24.59			
38		Ru-Snail	Unknown	24.55			
39		Ru-Snail	Unknown	24.58			
40		Ru-GAPDH	Unknown	11.74			
41		Ru-GAPDH	Unknown	11.79			
42		Ru-GAPDH	Unknown	11.79			
49		Ru-E-Cad°	Unknown	24.58			
50		Ru-E-Cad°	Unknown	24.60			
51		Ru-E-Cad°	Unknown	24.65			
64		Ru-GAPDH°	Unknown	12.17			
65		Ru-GAPDH°	Unknown	12.28			
66		Ru-GAPDH°	Unknown	12.24			

-

Legend:
NEG (NTC) - Sample cancelled due to NTC Threshold.
NEG (R. Eff) - Sample cancelled as efficiency less than reaction efficiency threshold.

This report was generated by Rotor-Gene Q Series Software 2.3.1 (Build 49)
Copyright ©2013 QIAGEN GmbH. All Rights Reserved.

Figure S4. Report RT-qPCR.
